# Chronic kidney diseases and the risk of colorectal cancer: A systematic review and meta-analysis

**DOI:** 10.1080/2090598X.2023.2225315

**Published:** 2023-06-20

**Authors:** Ahmad R. Al-Qudimat, Mohamed B. Al Darwish, Saif B. Altahtamouni, Kalapan Singh, Raed M. Al-Zoubi, Omar M. Aboumarzouk, Abdulla Al-Ansari

**Affiliations:** a Surgical Research Section, Department of Surgery, Hamad Medical Corporation, Doha, Qatar; b Department of Public Health, QU-Health, College of Health Sciences, Qatar University, Doha, Qata; c Department of Nursing, Hamad Medical Corporation, Doha, Qatar; d College of Pharmacy, QU Health, Qatar University, Doha, Qata; e Department of Chemistry, Jordan University of Science and Technology, Irbid, Jordan; f College of Medicine, Qatar University, Doha, Qatar; g School of Medicine, Dentistry and Nursing, The University of Glasgow, Glasgow, UK

**Keywords:** Chronic kidney disease, colorectal, cancer, CKD, Risk

## Abstract

**Objective:**

We conducted this review to offer a comprehensive search and up-to-date overview of the currently available information about the probability risk of colorectal cancer among chronic kidney disease patients.

**Method:**

We performed a systematic review and meta-analysis following Preferred Reporting Items for Systematic Reviews (PRISMA) and meta-analysis guidelines. We identified, reviewed, and extracted from Scopus, PubMed, EMBASE, and Komaki Databases for research publications on chronic kidney disease and colorectal cancer published between February 2016 and January 2023. We meta-analyzed the prevalence of colorectal cancer with chronic kidney disease. We ran a random effect meta-regression. Risk-of-bias assessment was evaluated using the Newcastle-Ottawa Scale. The systematic review was registered with PROSPERO (CRD42023400983).

**Results:**

The risk of CRC in chronic kidney diseases was reported in 50 research studies, which included 4,337,966 people from 16 different countries. SIR of CRC was obtained from 14 studies and showed a significant relationship between CRC with CKD patients, with a pooled SIR of 1.33; 95% CI (1.30–1.36), with higher heterogeneity (Q = 121.82, *P* < 0.001, and I^2^ = 86.9%). Metaregression showed that there was no significant correlation between the risk of CRC and the proportion of males or age.

**Conclusion:**

Overall, this study shows that patients with chronic kidney disease have a significantly increased risk of colorectal cancer. More studies with larger sample sizes, and robust surveillance are needed.

## Introduction

Chronic Kidney Disease (CKD) has always been one of the major chronic diseases worldwide as its prevalence is increasing in many countries such as the United States where it was 10% during the 1990s and that number went up to around 13% in 2004 [1,2]. Additionally, the constant decline of renal function and the Glomerular Filtration Rate (GFR) have been closely associated with cardiovascular diseases [3] as well as having multiple causes of mortality [4]. Moreover, it was found that the incidence of cancer was higher in patients with CKD than those who do not have it [5], this also applies to patients who received kidney transplants and had a cancer incidence ranging from 1.9% to 18% [6], and it is also suspected that immunosuppression is one of the possible explanations to the increased risk of malignancy in transplant patients [7]. With the recent advancements made in the field of transplantation like modern surgical techniques and immunosuppressive therapy, the survival rates of these patients have increased substantially. Therefore, in the long run, developing malignancy will be a major source of morbidity and mortality in these patients due to the increased life expectancy [8].

Colorectal cancer (CRC) is the third most common cancer diagnosed worldwide [9], which affects all races and is generally seen in elderly people [10]. However, early detection and treatment of CRC specifically in its early stages can significantly lower its morbidity and mortality with a chance of being cured, which makes early screening for the disease and determination of the risk factors very essential [11]. This applies to CKD patients who are more prone to cancer than the general population. Many studies explored the association between the two, one of those was a cross-sectional study done by Collins et al. in Australia, which concluded that patients who received kidney transplants had a higher prevalence of CRC than the general population and a screening tool, which is the fecal hemoglobin test that had a low sensitivity, which can lead to missing significant lesions that could turn cancerous. Thus, it is very important to develop surveillance guidelines for malignancy screening in patients with CKD [12].

Therefore, we are conducting this systematic review to expand further on the risk of developing colorectal cancer in patients with CKD and kidney transplant recipients and to determine if there is a significant association between the two, which would necessitate the application of more appropriate screening measures in patients with impaired renal function.

## Method

### Search strategy and selection criteria

For this review, studies were included if they met the following criteria: (1) population-based cohort studies evaluating the risk of colorectal cancer in CKD, (2) included SIRs and at least one complication, (3) included mortality information, and (4) accepted or published in English that could be extracted from scientific databases. Studies were excluded for the following reasons: (1) sampling of non-CKD, (2) could not extract or estimate SIRs and 95% CIs from the article, (3) studies that could not be extracted from the databases, and (4) lack of data availability.

Four e-databases were searched including PubMed, Embase, Scopus, and Cochrane Library. We were restricted to using key terms as follows: ‘Chronic kidney disease’ OR CKD OR ‘chronic kidney injury’ OR proteinuria OR albuminuria OR ‘glomerular filtration rate’ OR GFR OR ‘kidney disease’ OR ‘chronic kidney failure’ OR ‘chronic renal insufficiency’ AND ‘Colorectal cancer’ OR ‘colorectal carcinoma’ OR ‘colon cancer’ OR ‘rectal cancer’ OR ‘colorectal adenocarcinoma’ OR ‘familial colorectal cancer’ OR ‘colorectal lymphoma’ OR ‘colorectal neoplasms’.

### Data analysis and quality assessment

Two authors (M.B.D. and S.B.T.) independently screened and extracted the articles, by title and abstract, and then by full text to decide on the included studies. Any differences between reviewers at each stage of the screening process were discussed, and a consensus decision for eligibility and inclusion was made for all articles. All data items were checked by the author (A.R.A.). Newcastle- Ottawa Scale was used for the Quality appraisal for each article (A.R.A. and S.B.T.). The risk of bias assessment was performed separately for each study.

### Outcome assessment

The primary outcome for this review was to measure the colorectal cancer incidence and prevalence reported as SIR among different CKDs.

### Statistical analysis

To compare the risk of colorectal cancer among chronic kidney conditions, a random-effects meta-analysis was used. We assessed the presence of heterogeneity across studies using the I^2^ statistic, which measures the percentage of variability that can be attributable to between-study differences. Little heterogeneity, moderate heterogeneity, and high heterogeneity are all denoted by I^2^ values of 25%, 25% to 75%, and > 75%, respectively [13]. Statistical Q was also employed to assess the presence of heterogeneity between studies, and a P-value of less than 0.05 was taken as proof of statistically significant heterogeneity. We used the Begg and Egger test and funnel plots to depict potential asymmetries between studies to evaluate the possibility of a small-study effect or publication bias [14].

To investigate potential sources of heterogeneity, such as subject type, sex, age, and region, we performed subgroup meta-analysis or meta-regression. The 95% confidence interval (CI) of the regression coefficient was obtained throughout the meta-regression process. P-values that were < 0.05 were considered statistically significant. All statistical tests were two-sided.

## Results

### Study characteristics

**Figure 1.**
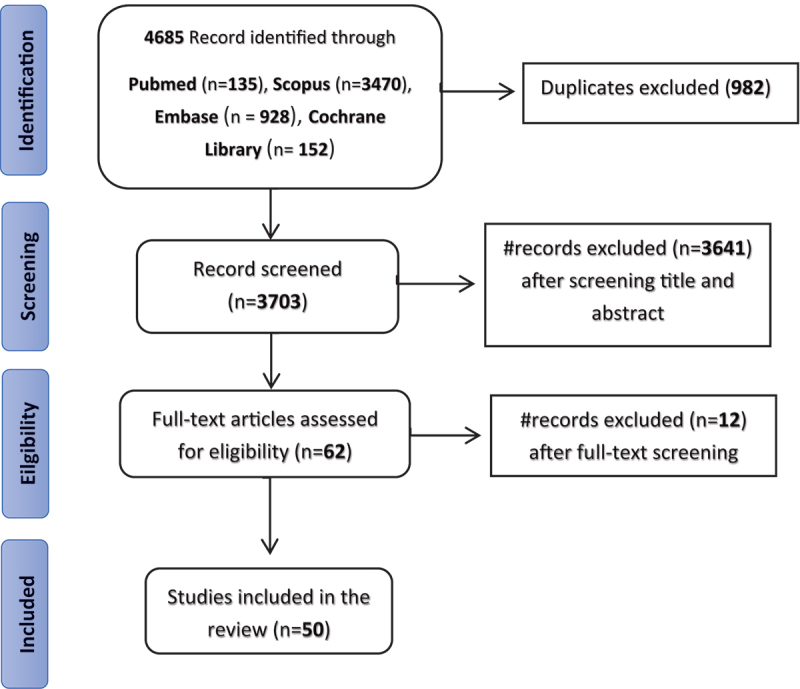
PRISMA diagram of literature search.

A total of 4,337,966 patients from 19 different countries, 3 from Japan, 5 from Australia/New Zealand, 2 from Canada, one from Finland, 2 from Hong-Kong (China), one from Egypt, 1 from Ireland, 5 from Italy, 7 from Korea, 1 from Malaysia, 1 from the Netherlands, 1 from Poland, 1 from Sweden, 1 from Portugal, 2 from Spain, 8 from Taiwan, 4 from the UK, and 4 from the USA, were included (Figure 1).

The studies were published from 1997 to 2022. Forty-six studies were retrospective, two were cross-sectional, one was case-control, and one was a matched cohort. Among the studies, 35 (70%) studies reported kidney transplants, 10 (20%) reported CKD, 3 (6%) reported dialysis and 2 (4%) reported the pre-dialysis patients. Table 1 provides an overview of the features and results of the listed research. Sixteen studies reported the median age to be 43–64, and 27 studies reported mean± SD ranging from 38.9 to 62.5 (Table 1).

**Table 1.** Summary of relevant studies.

Author (Years)	Study design	Country	No. of patients	Age mean±SD	% of Male	Prevalence of CRC	Newcastle-Ottawa Score
Collett, et al. (2010) [15]	Retrospective	UK	25104	NA	NA	0.72	7 (S:3, C:2, O:2)
van Leeuwen, et al. (2010) [16]	Retrospective	Australia	7809	43	59	NA	7 (S:3, C:2, O:2)
Wisgerhof, et al. (2011) [17]	Retrospective	Netherlands	1906	43.9	61.6	0.52	6 (S:2, C:2, O:2)
Cheung et al (2012) [18]	Retrospective	China	4895	43.7± 12.6	58.6	0.59	7 (S:3, C:2, O:2)
Li, et al. (2012) [19]	Retrospective	Taiwan	4716	44.1±12.4	52.5	0.25	8 (S:4, C:2, O:2)
Hall, et al (white) (2013) [20]	Retrospective	USA	49827	47	NA	0.26	6 (S:3, C:2, O:1)
Hall, et al (black) (2013) [20]	Retrospective	USA	20678	45	NA	0.23	6 (S:3, C:2, O:1)
Hall et al (hispanic) (2013) [20]	Retrospective	USA	17390	43	NA	0.13	6 (S:3, C:2, O:1)
Piselli, et al. (2013) [21]	Retrospective	Italy	7217	NA	64.2	0.29	6 (S:2, C:2, O:2)
Horie, et al. (2019) [22]	Retrospective	Japan	273	56	65	0.73	6 (S:3, C:0, O:3)
Kwon, et al. (2019) [23]	Retrospective	Korea	48315	NA	57	1.17	9 (S:4, C:2, O:3)
Lee, et al. (2018) PD [24]	Retrospective	Taiwan	35928	57.5 ± 11	55.1	0.06	9 (S:4, C:2, O:3)
Lee, et al. (2018) HD [24]	Retrospective	Taiwan	35928	58.5 ±11.7	55.1	0.17	9 (S:4, C:2, O:3)
Oh, Hyung Jung et al. (2018) [25]	Retrospective	Korea	35443	NA	49.7	1.37	7 (S:4, C:0, O:3)
Collins et al. (2012) [12]	Cross-sectional	Australia	229	61.5 ± 6.9	66	12.66	5 (S:3, C:0, O:2)
Matsuoka et al. (2022) [26]	Retrospective	Japan	2745296	NA	55.9	0.01	8 (S:4, C:1, O:3)
Saumoy et al. (2016) [27]	Retrospective	USA	140	57 ± 7.7	69	0.00	8 (S:4, C:1, O:2)
Wu et al. (2013) [28]	Matched	Taiwan	15975	NA	52	0.73	8 (S:4, C:1, O:3)
Balhareth et al. (2018) [6]	Retrospective	Ireland	4230	62.5 ± 1.9	NA	0.78	6 (S:3, C:0, O:3)
Wang et al. (2019) [29]	Retrospective	Taiwan	46063	61.84±12.6	45	0.29	9 (S:4, C:2, O:3)
Stewart et al. (1997) [30]	Retrospective	UK/Germany	62088	NA	NA	0.11	5 (S:2, C:2, O:1)
Kyllonen et al. (2000) [31]	Retrospective	Finland	2890	41.5	59.5	0.45	7 (S:3, C:2, O:2)
Adami et al. (2003) [32]	Retrospective	Sweden	5004	46	61	0.78	7 (S:3, C:2, O:2)
Vajdic et al. (2006) [33]	Retrospective	Australia	28855	50	56.7	1.18	7 (S:3, C:2, O:2)
Villeneuve et al. (2007) [34]	Retrospective	Canada	11155	NA	63.2	0.46	7 (S:3, C:2, O:2)
AlAmeel et al. (2015) [35]	Retrospective	Canada	169	57.9	61	0.59	4 (S:2, C:0, O:2)
Daud et al. (2022) [36]	Cross-sectional	Malaysia	171	NA	56.7	1.17	5 (S:3, C:0, O:2)
Lizakowski et al. (2018) [37]	Retrospective	Poland	3069	48.8	NA	0.52	6 (S:3, C:0, O:3)
Lin MY et al. (2015) [38]	Retrospective	Taiwan	47037	58.2±15.1	47.2	0.40	6 (S:3, C:1, O:2)
Butler, Anne M et al (2015) [39]	Retrospective	USA	456996	69.5	52	0.91	6 (S:3, C:0, O:3)
Tessari, G et al (2013) [40]	Retrospective	Italy	3537	NA	65	0.42	8 (S:3, C:2, O:3)
Kim, Ji Hyun et a l(2014) [41]	Retrospective	Korea	2365	39.4±11.8	61	0.38	8 (S:3, C:2, O:3)
Aguiar, B et al (2015) [42]	Retrospective	Portugal	2353	44.44±14.12	67	0.68	8 (S:3, C:2, O:3)
Bakr, M A et al (1997) [43]	Retrospective	Egypt	950	NA	NA	0.11	6 (S:2, C:1, O:3)
Chung, Mu-Chi et al (2014) [18]	Retrospective	Taiwan	4350	NA	52	0.32	7 (S:3, C:1, O:3)
Johnson, Erik E et al (2007) [44]	Retrospective	USA	4794	NA	25	0.00	7 (S:3, C:1, O:3)
Kato, Taigo et al(2016) [45]	Retrospective	Japan	750	38.9±10.5	60	0.67	8 (S:4, C:1, O:3)
Kwon, Jee Hye et al (2015) [46]	Retrospective	Korea	248	52.6±12.3	62.5	1.61	7 (S:3, C:1, O:3)
Navarro et al. (2008) [47]	Retrospective	Spain	1017	NA	NA	0.49	8 (S:3, C:2, O:3)
Privitera et al. (2021) [48]	Case-control	Italy	160	49.2±8.15	59.3	2.50	7 (S:4, C:1, O:2)
Rosales et al (2020) [49]	Retrospective	Australia	17628	NA	NA	0.00	7 (S:4, C:1, O:3)
Rossetto et al. (2015) [50]	Retrospective	Italy	636	NA	NA	2.04	6 (S:3, C:0, O:3)
Au et al. (2018) (dialysis) [51]	Retrospective	Australia	52936	NA	59	0.00	7 (S:3, C:1, O:3)
Au et al. (2018) (transplant) [51]	Retrospective	Australia	16820	NA	60.5	0.00	7 (S:3, C:1, O:3)
Buxeda et al. (2019) [52]	Retrospective	Spain	925	47.9±14.2	62.8	0.54	7 (S:3, C:1, O:3)
Chinnadurai et al. (2019) [53]	Retrospective	UK	2952	NA	62.1	1.96	7 (S:3, C:2, O:2)
Gioco et al. (2019) [54]	Retrospective	Italy	535	48±22.3	48	0.37	7 (S:3, C:2, O:2)
jackson-spence et al. (2018) [55]	Retrospective	UK	19883	47.55±13.69	61.47	0.39	9 (S:4, C:2, O:3)
Jung et al. (2022) [56]	Retrospective	Korea	12634	NA	65.8	0.00	7 (S:3, C:1, O:3)
Kao et al. (2018) (mTOR inhibitors users) [57]	Retrospective	Taiwan	828	44.8±11.5	57.6	0.48	8 (S:3, C:2, O:3)
Kao et al. (2018) (mTOR inhibitors non-users) [57]	Retrospective	Taiwan	3735	47±11	52.7	0.72	8 (S:3, C:2, O:3)
Liu et al. (2022) [58]	Retrospective	China	811	41.2±11.1	70.4	0.12	8 (S:3, C:2, O:3)
Myung et al. (2018) [59]	Retrospective	Korea	14382	58.22±12.15	56.5	0.88	8 (S:3, C:2, O:3)
Park et al. (2019) [60]	Retrospective	Korea	471,758	NA	51.5	0.61	8 (S:3, C:2, O:3)
Yeh et al. (2020) [61]	Retrospective	Taiwan	5038	44.4±12.3	52.9	0.40	7 (S:3, C:1, O:3)

Newcastle-Ottawa Score: good quality (up to 3), fair quality (2), poor quality (0 or 1).

### Risk of colorectal cancer among patients with chronic kidney diseases

**Figure 2.**
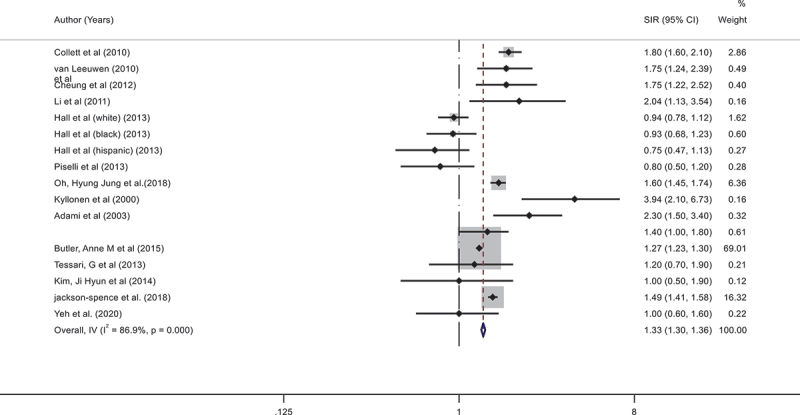
Forest plot of Incidence rate colorectal cancer with chronic kidney disease.

Fourteen studies reported patients with colorectal cancer with chronic kidney disease. The pooled SIR was 1.33; 95% CI (1.30–1.36) demonstrates a significantly increased risk of colorectal cancer with higher heterogeneity (Q = 121.82, *P* < 0.001, and I^2^ = 86.9%) (Figure 2). From this plot, we can see that the incidence rate of colorectal cancer among the included studies ranges from a minimum of 0.75 (95% CI: 0.47–1.13) [20] to a maximum of 3.94 (95% CI :2.10–6.73) [31].

**Figure 3.**
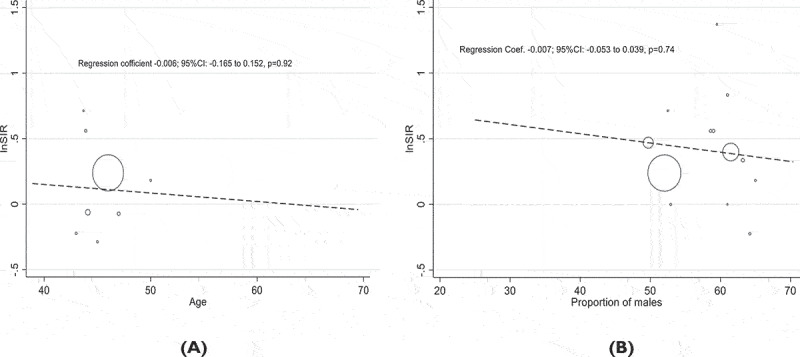
(a). Meta-regression of the Age, (b). The proportion of males (%) and risk of colorectal cancer with kidney disease.

The association between age, the percentage of men, and the risk of colorectal cancer with kidney disease was examined using meta-regression. Age and the risk of colorectal renal disease were not significantly correlated, according to our findings. The residual variance owing to heterogeneity I^2^ was 78.0%. In terms of the proportion of males, no association was found β=−0.007: 95%CI (−0.053 to 0.039) with residual variance owing to heterogeneity I^2^ = 82.3% (Figure 3).

**Figure 4.**
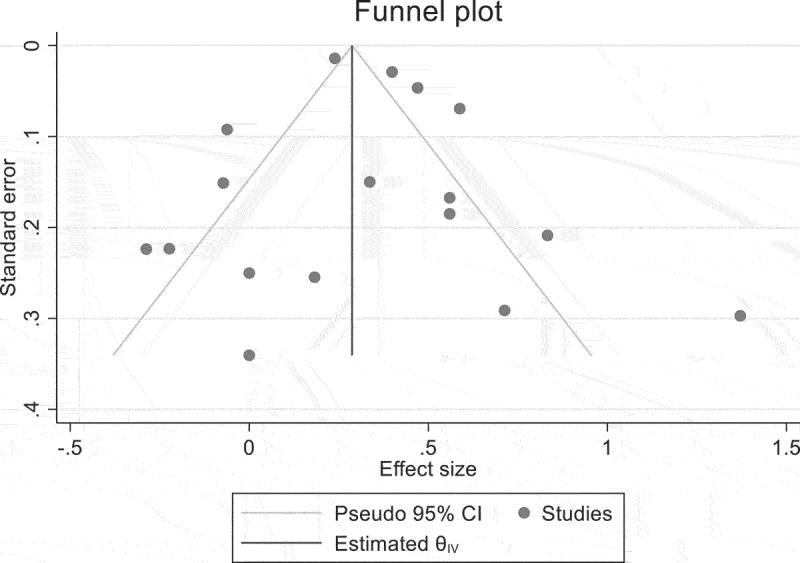
Funnel plot of detecting publication bias.

In the current meta-analysis, no small study effect was found (Figure 4); we utilized Egger’s linear regression test to determine the publication bias, which was 0.15 (*p* = 0.88), which is not statistically significant. No concrete proof of publishing bias exists.

## Discussion

In this review, we conducted a systematic review and meta-analysis on all the relevant articles we were able to extract from the literature to explore the risk of developing Colorectal Cancer (CRC) in patients with Chronic Kidney Disease (CKD). Our results demonstrated that there is in fact a significantly increased risk of developing colorectal malignancy in patients with chronic renal disease.

Multiple observational retrospective studies have explored the risk of developing malignancies in patients with chronic renal disease who were undergoing dialysis or patients who underwent kidney transplant. These studies have consistently found that these patients were at a higher risk of developing several kinds of malignancies like solid organ cancers, melanoma, and lymphoma [23,37,62]. Additionally, some articles studied the risk of developing colorectal neoplasia specifically in kidney transplant patients and concluded that there was an increased risk of being diagnosed with an advanced colorectal neoplasia (IRR, 1.52; 95% CI: 1.43–1.61) (13%, 95% CI: 9% to 18%) [12,23,37]. This is consistent and comparable to our result; the pooled SIR was 1.33; 95% CI: (1.30–1.36) and demonstrated a significantly increased risk of colorectal cancer. Moreover, we also conducted a subgroup analysis to determine the effect of geographic location, it showed that geographical region (for north America: SIR: 1.07; 95% CI:0.87, 1.31, *p* = 0.0.061) and Europe SIR 1.66; 95%CI:1.12, 2.47. *p* = 0.647) had a significant influence on colorectal cancer with chronic kidney disease. This could be explained by the number of studies performed in North America and Europe that could affect this outcome. We also performed a subgroup analysis to determine the gender differences in CRC incidence, and we concluded that percentage of males had a significant association between the effect of colorectal cancer with chronic kidney disease. However, male patients had a significantly higher risk of colorectal cancer with chronic kidney disease (the percentage of men > 50%; SIR: 1.50: 95% CI: (1.22, 1.83); the percentage of men is ≤50%: SIR: 1.60; and 95% CI: (1.46, 1.75); this result goes in line with multiple studies that reported similar outcomes in patients with or without CKD [63,64] (Table 2).

**Table 2.** Subgroup analysis of association between colorectal cancer and chronic kidney disease.

Subgroups	Study (n)	SIR (95%CI)	Heterogeneity test		
			Q	p-value	I^2^ (%)
Overall	17	1.33 (1.30–1.36)	121.82	0.000	86.9%
Geography					
*Australia*	1	1.75 (1.26, 2.43)	0.00	-	-
*North America*	5	1.07 (0.87, 1.31)	20.26	0.000	79.68
*Europe*	6	1.66 (1.12, 2.47)	29.59	0.000	95.08
*Asia*	5	1.58 (1.45, 1.72)	6.29	0.178	0.00
The percentage of men					
*≤50%*	1	1.60 (1.46, 1.75)	0.00	0.000	-
*>50%*	12	1.50 (1.22, 1.83)	60.07	0.001	94.86

The increased risk of developing colorectal neoplasia in CKD patients could be attributed to multiple predisposing factors such as the chronic inflammation. In addition, chronic kidney disease has been associated with the increased production of proinflammatory cytokines, which could be contributed to the state of chronic inflammation and a predisposing factor to multiple malignancies by inducing some mutations such as proneoplastic and angiogenesis and making cells more resistant to apoptosis [65]. Furthermore, it was suggested by Yu et al. that advanced stages of renal disease could potentially cause damage to the intestinal mucosa and precipitated in malignant and neoplastic transformations in these cells [66].

Immunosuppression is another proposed factor that increases the risk of developing multiple types of cancers especially in patients undergoing immunosuppressive therapy and after receiving organ transplants such as kidney transplant [33]. Additionally, some studies linked the risk of developing malignancy with the type of immunosuppressive agent, they found that patients treated with steroids and thiopurines had higher incidence rates of cancers in the epithelium of different organs including the colon, whereas patients who were treated with ciclosporins did not exhibit any increase in their risk of contracting malignancy [67,68]. This is consistent with our results where a large proportion of our sample was patients post-kidney transplant, and we demonstrated an increase in the risk of developing CRC. However, more studies should explore the relationship between immunosuppressive treatments and developing colorectal cancer.

## Limitation

Our study contained some limitations. First, only observational studies were included in this meta-analysis, even though all mentioned studies were of high quality and had low risk of bias. Furthermore, our meta-analysis did not provide sufficient details related to mortality due to the lack of information in the included studies. Moreover, we were unable to categorize patients with chronic kidney disease based on stages and severity because of insufficient data. Finally, the fact that sample size considerably varies between selected studies led to some heterogeneities.

## Conclusion

This meta-analysis of this study indicates that CKD is considered a high-risk population for the development of colorectal cancer, regardless of the stage of cancer. Additional studies with larger sample sizes are needed to confirm these findings.

## Data Availability

All data analyzed during this study are included in this article, and further inquiries can be directed to the corresponding author.

## Abbreviations

CKD: Chronic Kidney Disease
GFR: Glomerular Filtration Rate
CRC: Colorectal Cancer
SIR: Standardized Incidence Ratio
